# Cocaine‐Induced Apoplexy of a GH‐Secreting Pituitary Tumor

**DOI:** 10.1002/ccr3.70497

**Published:** 2025-05-12

**Authors:** Dalia Cuenca, Latife Salame‐Khouri, Daniela Shveid‐Gerson, Marco Antonio Alegría‐Loyola, Moises Mercado

**Affiliations:** ^1^ Department of Medicine American British Cowdray Medical Center Mexico City Mexico; ^2^ Department of Neurology Instituto Nacional de Ciencias Médicas y Nutrición Salvador Zubirán Mexico City Mexico; ^3^ Endocrine Research Unit, Hospital de Especialidades, Centro Médico Nacional Siglo XXI, IMSS Mexico City Mexico

**Keywords:** acromegaly, cocaine, growth hormone, pituitary apoplexy, tumor

## Abstract

A 25‐year‐old previously healthy man presents to the clinic with a severe headache after cocaine consumption. Physical examination revealed acromegaloid features, and the hormonal profile revealed an IGF‐1 of 308 ng/mL (40.8 nmol/L) (normal reference range 197–333 ng/mL; 17.8–45.6). An MRI showed a heterogeneous, cystic lesion of the pituitary gland with hematic contents, consistent with the diagnosis of a pituitary apoplexy. Considering the normal IGF‐1 level, the patient was classified to be in remission, which deems it unnecessary to initiate pharmacologic or surgical treatment. Remission of acromegaly after a pituitary apoplexy is an extremely rare event, and pituitary apoplexy after cocaine consumption is also anecdotal. We present the first case of remission of acromegaly after a cocaine‐induced pituitary apoplexy.

**Summary**

- Remission of acromegaly after pituitary apoplexy is an extremely rare event, and pituitary apoplexy after cocaine consumption is unknown.
- Herein, we present the first case of acromegaly remission after cocaine‐induced pituitary apoplexy.

## 1 Introduction

Pituitary apoplexy (PA) is an infrequent but life‐threatening endocrine emergency that results from the hemorrhagic infarction of the pituitary gland with or without a pituitary tumor. PA has a prevalence of 6.2 cases per 100,000 and an incidence of 0.17 per 100,000 people per year and is more frequent in men between 40 and 50 years of age [1]. A triggering factor was identified in 10%–40% of the cases. The most common precipitating causes include treatment with dopamine agonists, the use of hypothalamic‐releasing hormones in pituitary dynamic testing, angiography, orthopedic surgery, cardiac surgery, and head injury [1]. Cocaine is a vasoactive drug that causes cerebral vasoconstriction that can cause hemorrhagic infarction [2]. A few isolated reports of cocaine‐induced apoplexy of pituitary tumors have appeared in the literature; however, PA occurring in the context of a functioning adenoma is remarkably rare. Furthermore, amelioration of growth hormone (GH) hypersecretion due to hemorrhagic infarction of a somatotropinoma is theoretically possible but largely unheard [3, 4].

## 2 Case Presentation

A previously healthy 25‐year‐old man presented to the emergency room with a one‐week history of severe abrupt‐onset holocranial headache. The headache, which developed after inhaling cocaine, was accompanied by blurred vision, nausea, dizziness, and dysarthria, and was not relieved by acetaminophen or ketorolac. Upon specific questioning, he admitted being a frequent cocaine user and reported an increase in shoe size over the previous year. On physical examination, he was found to have orthostatic hypotension (blood pressure 90/50 mmHg seating, 70/40 mmHg standing), pulse 100 per minute and regular, temperature 36.5°C, acromegaloid habitus with evident macroglossia, prognathism, large nose, supraciliary arches, hands, and feet; visual fields by confrontation were normal.

## 3 Diagnostic Assessment

**FIGURE 1:**
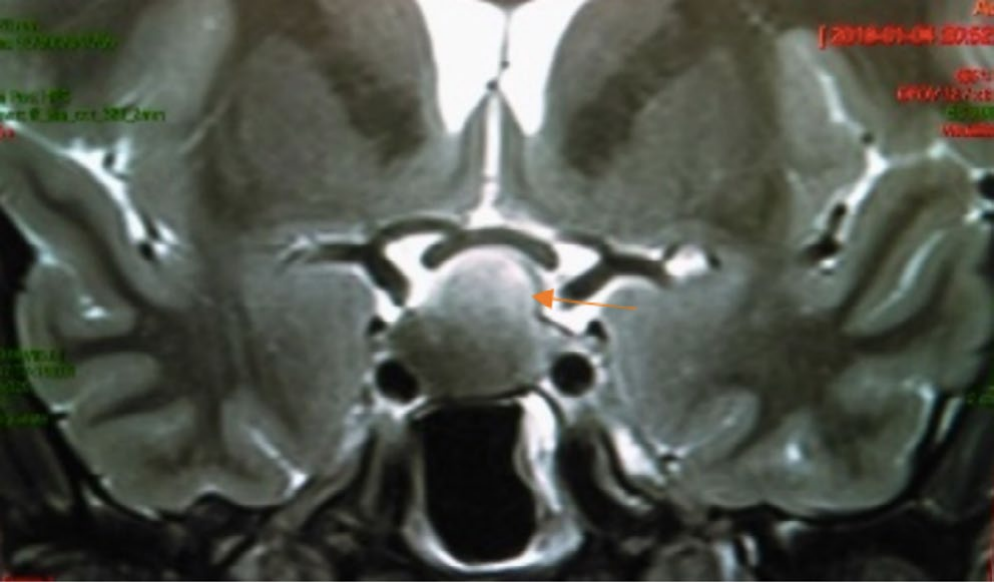
T2‐weighted MRI of the sellar region showing an heterogeneous, partially cystic pituitary lesion with evidence of recent hemorrhage, measuring 18.7 × 13.5 × 18.7 mm (arrow).

His hormonal profile included a morning cortisol of 5.1 g/dL (reference range 5–25 g/dL), adrenocorticotropic hormone (ACTH) 22 pg/mL (reference range 7.2–63 pg/mL), total testosterone of 364 ng/dL (reference range 300–1000 ng/dL), luteinizing hormone (LH) level 2.16 mIU/mL (reference range 0.57–12.07 mIU/mL), follicle‐stimulating hormone (FSH) level 4.5 (reference range 0.96–11.95 mIU/mL), thyroid‐stimulating hormone (TSH) 2.46 mIU/mL (reference range 0.3–4.2 mIU/mL), free T4 1.14 ng/dL (reference range 0.9–1.7 ng/dL), a prolactin level of 22.5 ng/mL (1.6–18.7 ng/mL) and this was corroborated with diluted prolactin, and an insulin‐like growth factor‐1 (IGF‐1) 308 ng/mL (reference range 197–333 ng/mL), and a GH after 75 g glucose tolerance test of 1.83 ng/mL (reference range < 0.3 ng/mL). Magnetic resonance imaging (MRI) of the sellar region showed a heterogeneous, partially cystic pituitary lesion with evidence of recent hemorrhage, measuring 18.7 × 13.5 × 18.7 mm (Figure 1). A diagnosis of pituitary tumor apoplexy was entertained so he was started on intravenous hydrocortisone, 100 mg every 8 h.

## 4 Treatment

The patient showed dramatic improvement after glucocorticoid replacement, and surgical treatment was deemed unnecessary. He was discharged home asymptomatic one week after admission, with prednisone 5 mg daily due to availability in our clinic, and during follow‐up, the treatment was switched to hydrocortisone.

## 5 Outcome and Follow‐Up

During follow‐up, the corticotropic axis recovered after two years and is currently without hormonal deficit. The patient was asymptomatic and had no clinical acromegaly; the clinical features regressed during follow‐up. Although his IGF‐1 level remained within the normal limits for age, his GH persisted mildly elevated and failed to suppress appropriately upon glucose loading, and he is currently on yearly follow‐up with hormonal work to detect early recurrence signs. An MRI of the pituitary was performed after the event, and it showed reduction in the tumor, with a residual mass of < 1 cm.

## 6 Discussion

PA develops in 2%–12% of patients with pituitary adenomas. Although PA has been reported in all types of pituitary tumors, the majority of patients harbor nonfunctioning pituitary macroadenomas. However, in more than 75% of cases, patients are unaware of the presence of pituitary tumors [1]. The basic pathophysiology of PA remains unclear, but the most popular theory involves a microcirculation imbalance between the tumor and the surrounding normal gland, whereby, as the former increases in size, it outgrows its already limited blood supply. Thus, the tumor has reduced blood flow despite its high metabolic demand compared to non‐tumoral pituitary tissue [5]. Most of the triggering factors associated with PA (dopamine agonists, hypothalamic releasing hormones, head trauma, and cardiac and orthopedic surgery) result in a further increase in tumor metabolic demand [1]. Therefore, tumors are vulnerable to disruption of the balance between metabolic demand and perfusion. Cocaine is a vasoactive drug associated with cerebrovascular diseases, both ischemic and hemorrhagic [6]. Vasoconstriction is followed by reperfusion of ischemic zones, which provokes hemorrhage associated with cocaine use [6]. The powerful vasoconstrictive effect of cocaine can further compromise the equilibrium, resulting in hemorrhagic infarction of the tumor [4].

Cocaine has been implicated as a causal factor of PA in one patient with nonfunctioning pituitary adenoma [7]. Two additional cases of panhypopituitarism secondary to chronic cocaine consumption have been described [8, 9]. To our knowledge, this is the first case report of remission of acromegaly due to PA in a GH‐secreting tumor after acute cocaine inhalation.

Since 1975, 29 cases of acromegaly remission have been reported following pituitary apoplexy [4]. Although our patient experienced drastic clinical improvement after the apoplectic event and his IGF‐1 level decreased to normal, he cannot be in full remission since his basal and glucose‐suppressed GH levels persisted slightly elevated seven years after the acute event [10, 11]. It is difficult to predict whether the somatotropic axis will eventually normalize.

The important aspects of the management of this patient are worth mentioning. First, the patient was treated conservatively with acute glucocorticoid replacement and hemodynamic stabilization, with indefinite postponement of surgical management. Although this conservative strategy may be debatable, the patient's course and evolution proved correct. However, the lack of complete biochemical remission of acromegaly, at least in theory, warns us of the possibility of future recurrence.

## Author Contributions

**Dalia Cuenca:** conceptualization, methodology, writing – original draft. **Latife Salame‐Khouri:** conceptualization, methodology, writing – original draft. **Daniela Shveid‐Gerson:** conceptualization, writing – original draft, writing – review and editing. **Marco Antonio Alegría‐Loyola:** conceptualization, investigation, writing – review and editing. **Moises Mercado:** conceptualization, data curation, methodology, writing – review and editing.

## Disclosure

The authors have nothing to report.

## Ethics Statement

Ethics approval is not required for this study in accordance with local or national guidelines. Written informed consent was obtained from the patient for publication of the details of their medical case and any accompanying images.

## Conflicts of Interest

The authors declare no conflicts of interest.

## Data Availability

All data generated or analyzed during this study are included in this article and its online Supporting Information. Further enquiries can be directed to the corresponding author.
